# Acorn-related acquired pseudomelanosis in Calabrian black pigs

**DOI:** 10.1186/s12917-019-1934-5

**Published:** 2019-06-04

**Authors:** Giovanni Lanteri, Jessica Maria Abbate, Carmelo Iaria, Daniele Macrì, Vincenzo Ferrantelli, Fabio Marino

**Affiliations:** 1 Department of Veterinary Sciences, University of Messina, 98168 Messina, Italy; 2 Department of Chemical, Biological, Pharmaceutical and Environmental Sciences, University of Messina, 98166 Messina, Italy; 3 Istituto Zooprofilattico Sperimentale della Sicilia, Via Gino Marinuzzi 3, 90129 Palermo, Italy

**Keywords:** Acorn, Pseudomelanosis, Quercus, Swine, Thesaurismosis

## Abstract

**Background:**

Melanosis of lymph nodes in black pigs has generally been related to regression of congenital melanoma and, occasionally, to ingestion of acorns. The aim of this manuscript is to confirm the hypothesis of a possible acquired acorn-related pseudomelanosis in the *Nero Calabrese pig*, a swine breed belonging to the group of Italian native breeds and whose coverage area corresponds to the region of Calabria, southern Italy. This pig is characterized by slow-growing subjects, producing, however, high quality meat suitable for the production of sausages and fine hams. The study was carried out on 142 normally slaughtered pigs. All organs were examined. Lymph nodes and intestine (jejunum) were sampled. Histochemistry was performed on deparaffinized histological sections to identify the cell types involved and to characterize the pigment stored. To further confirm the pigmentation disorder, immunohistochemistry was carried out. Total phenolic substances were identified in acorns through the use of a biochemical reaction.

**Results:**

Lymph node pigmentation appears directly related to acorn ingestion, with a higher incidence in the group which was 70% natural fed (acorn of *Quercus virgiliana*). Moreover, findings obtained revealed how different amounts of phenolic substrates present in *Q. virgiliana* and *Q. ilex* acorns can influence the incidence of such exogenous pigmentation.

**Conclusion:**

The findings obtained in this study confirm the acquired nature of the melanin-like pigmentation detected in lymph nodes from acorn-fed swine. Acquired pigmentation must be differentiated from true melanosis as well as from melanosis related to tumor regression of congenital melanoma. This thesaurismosis can be proposed as a marker of wellbeing and quality, confirming that the pigs have been bred and fed in natural conditions.

**Electronic supplementary material:**

The online version of this article (10.1186/s12917-019-1934-5) contains supplementary material, which is available to authorized users.

## Background

Pathological color modifications in tissue include a vast number of different categories; pathological pigmentations are identified as deposition of substances with a specific coloration in cells and tissues; exogenous pigments are introduced from the external environment and are stored in cells and in tissues; endogenous pigmentations are products of metabolism; thus, their appearance is due to metabolic disturbance. Endogenous pigmentations are caused by colorant substances such as lipofuscin, melanin and the group of hem pigments, derived from blood pigment hemoglobin [1]. The most important and known endogenous pigmentation is melanosis, a grey-black pigmentation found in different organs due to melanin storage [2].

Melanin (in Greek melas = black), an autochthonous intracellular pigment, is characterized by a brown-black color.

It is produced by tyrosine oxidation, which represents the structural basis of its molecule, tyrosinase (with Apo enzyme containing copper) through deoxy phenylalanine (DOPA) and indolquinone. Melanin is produced by specific cells, melanocytes, whose precursors - melanoblasts (non-pigmented cells) - derive from the neural crest [1, 3]; melanocytes can release pigment to other cells as opposed to melanophages and keratinocytes, which are capable of storing but not of synthetizing it [3].

Melanin production may be influenced by neuroendocrine, as well as exogenous, factors (X-rays, UV rays, arsenic, phenolic substances, etc.). Phenols can influence polymerization of quinones, derived from phenol oxidation, which assume a dark discoloration [4].

Melanin contained in melanosomes is collected from pigmented cells of the epidermis, the hair matrix, intestine mucosa and pia mater of some specific tracts of the brain (substantia nigra), and the choroid.

Several pathological modifications linked to melanin can occur: some tumors, e.g. melanoma, melanosis, acanthosis nigricans, skin anomalies in human beings, skin hyperpigmentation associated to hyperadrenocorticism, in the rare human hereditary disease known as Dubin-Johnson syndrome; in mutant Corredale sheep, in which an excretory defect of the liver produces an accumulation of melanin pigment in hepatocytes [5]. Pathological changes related to melanin can be due to a perturbation of those processes related to production, transport and dispersion of pigments [4].

Melanosis of lymph nodes in some black pigs, such as Sinclair, Duroc-Jersey, Hormel, Vietnamese pot-bellied and their cross-breeds, has also been related to regression of congenital melanoma [6–9].

In general, melanosis represents an unusual finding at the slaughterhouse [10] and to date, except in solipeds, it is not considered a disease to notify, although carcasses showing extensive melanosis are condemned. If the condition is localized, only the affected organ or part of the carcass needs to be condemned according to FAO recommendations [11]. Recent investigations, performed at slaughterhouses on the autochthone swine breed Nero Siciliano pig reared free-range (in plein-air), have shown an increased incidence of melanosis [4, 12].

The Nero Calabrese pig breed is characterized by slow growing subjects, producing high-quality meat suitable for the production of sausages and fine hams, with meat pH tending to decrease more quickly compared to the modern depigmented breeds. Monoinsature and polyinsature fatty acids of the omega-3 and omega-6 series are more present in the meat of the black pigs reared free range as compared to those reared intensively. Another advantage derives from the fact that animals, because of their rusticity, can be farmed free range all year and can adapt very well to any possible feed, including food derived from grazing. In such a kind of farming system, animals live in a large closed off woodland area, where they can feed on natural pabulum, generally composed of acorns, tubers, roots, etc. Commercial food is daily provided only as an integrative supplement, thus reducing the environmental impact of these foodstuffs.

The aim of the present paper was to verify the possible acquired pathogenesis for a melanin-like pigmentation in Nero Calabrese pigs, by macroscopic, histological, immuno-histochemical and biochemical exams. Moreover, considering the high frequency in the Calabrian region of holm oak and quercus acorns being used as swine feed, it was possible to demonstrate how a different phenolic substrate available in different types of acorns may influence the incidence of exogenous pigmentation.

## Results

Results obtained by macroscopic and microscopic evaluation performed on pig specimens have been included in Table 1. Briefly, subjects which had been fed this food for a longer period of time/higher quantity showed the highest presence of phenolic substrate in quercus acorn and an increased incidence of the pigmentation. Moreover, groups fed with *Q. virgiliana* showed a greater melanosis prevalence than those fed with *Q. ilex*. On the other side, no significant different labelling was demonstrated by the histochemistry and immunohistochemistry between the different positive groups. In details: Group A (34 subjects, 70% natural fed/present in grazing [quercus acorn “*Quercus virgiliana*”, roots, tubers] + 30% commercial fed) showed pseudomelanosis of lymph nodes in 34/34 subjects (100% prevalence); group B (42 subjects, 40% natural fed/present in grazing [quercus acorn “*Quercus virgiliana*”, roots, tubers] + 60% commercial fed) showed a positivity of 36/42 subjects (85.71%); group C (35 subjects, 70% natural fed/present in grazing [holm oak acorn “*Quercus ilex*”, roots, tubers] + 30% commercial fed). showed a positivity of 28/35 subjects (80%); group D (31 subjects, 40% natural fed/present in grazing [holm oak acorn “*Quercus ilex*”, roots, tubers] + 60% commercial fed) showed a positivity of 23/31 (74.19); group E (29 subjects, 100% commercial fed) showed no pseudomelanosis (0%). Statistical analysis highlighted a significant difference in pseudomelanosis prevalence among groups. In particular, group A (total fed with Q. virgiliana) evoked a greater pseudomelanosis prevalence than groups B (χ2 189.50; *P* < 0.0001), C (χ2 7.57; *P* = 0.003), D (χ2 10.01; *P* = 0.0008) and E (χ2 63.00; P < 0.0001). Chi square test showed a significant higher pseudomelanosis prevalence in groups B (χ2 50.42; *P* < 0.0001), C (χ2 41.20; *P* < 0.0001) and D (χ2 50.20; *P* < 0.0001) compared to group E.

**Table 1:** Prevalence of pseudomelanosis in examined subjects

	Positive	Examined	Prevalence (%)
GROUP A	34	34	100
GROUP B	36	42	85.71
GROUP C	28	35	80
GROUP D	23	31	74.19
GROUP E	0	29	0

**Fig. 1:**
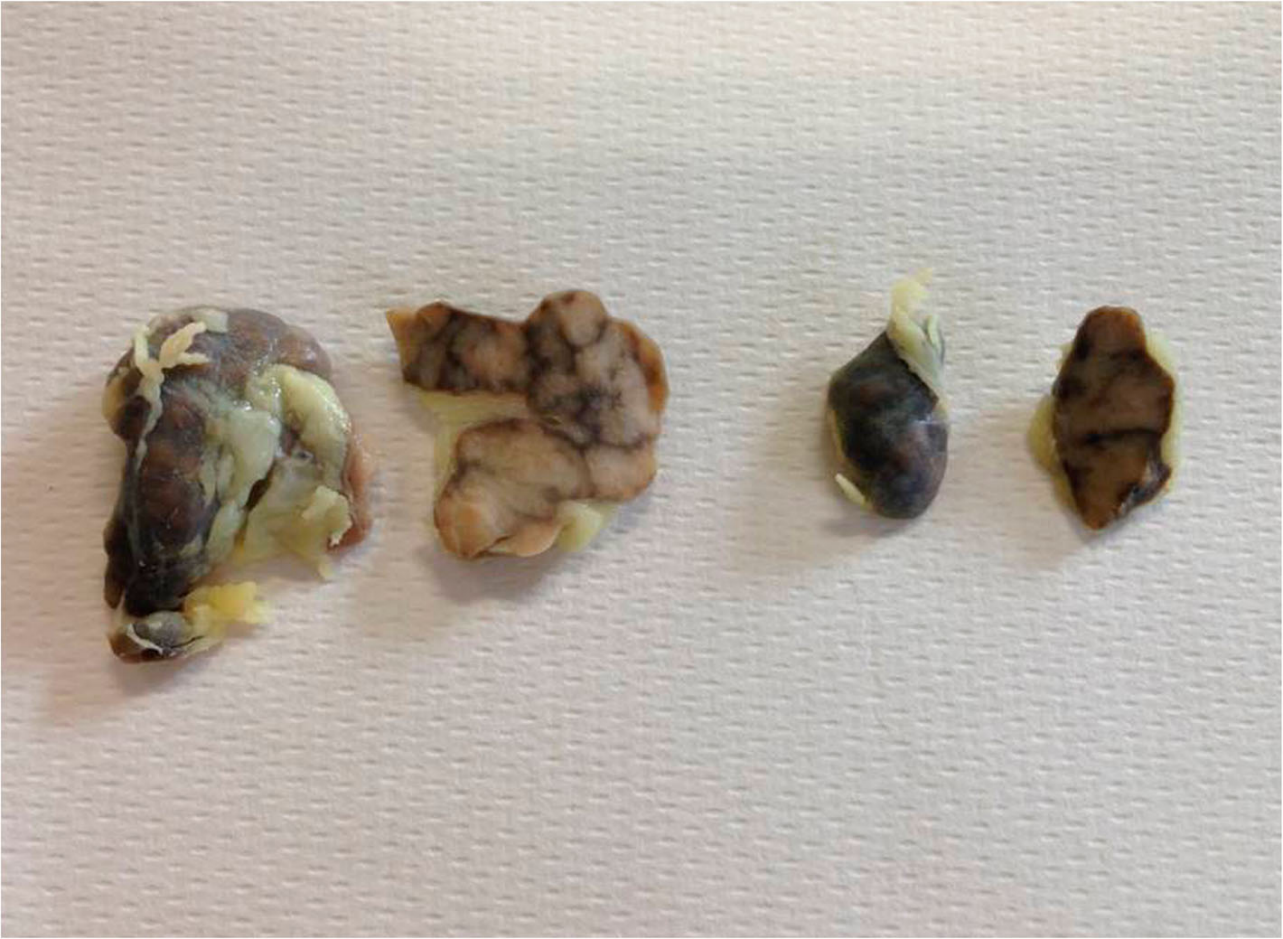
Lymph nodes: Macroscopic evidence of brown-black tissue discoloration

Macroscopic exam carried out on lymph nodes did not show any modification in shape and volume; the only evident feature was the strong black-brownish discoloration of the tissue which, in all the positive cases observed, involved both cortical and medullar portions (Fig. 1). None of the control animals showed any pigmentation in lymph nodes.

**Fig. 2:**
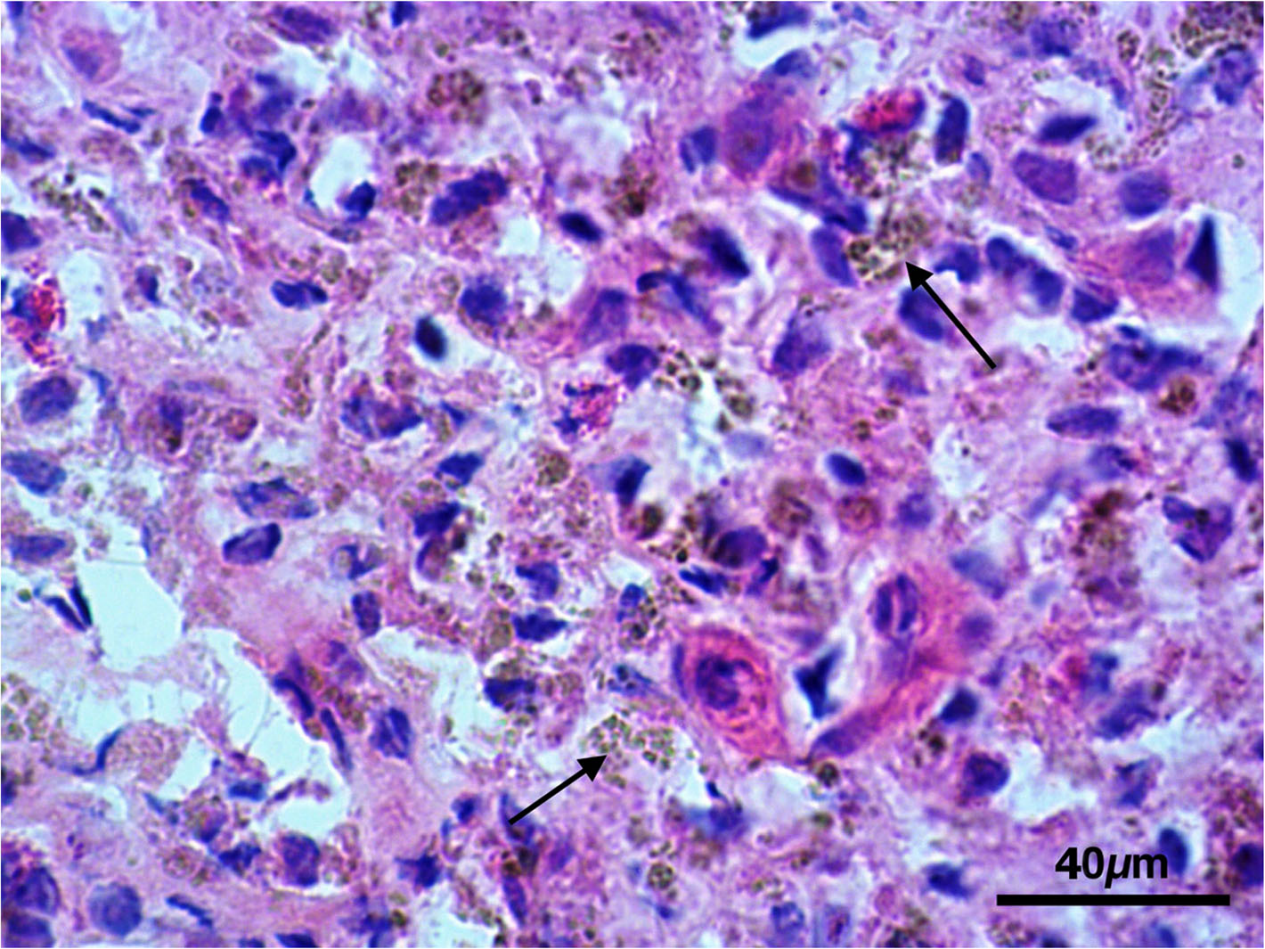
Lymph nodes: Lymph node tissue sections shown a macrophage infiltrate containing a granular brownish pigment (Arrows) (H&E)

**Fig. 3:**
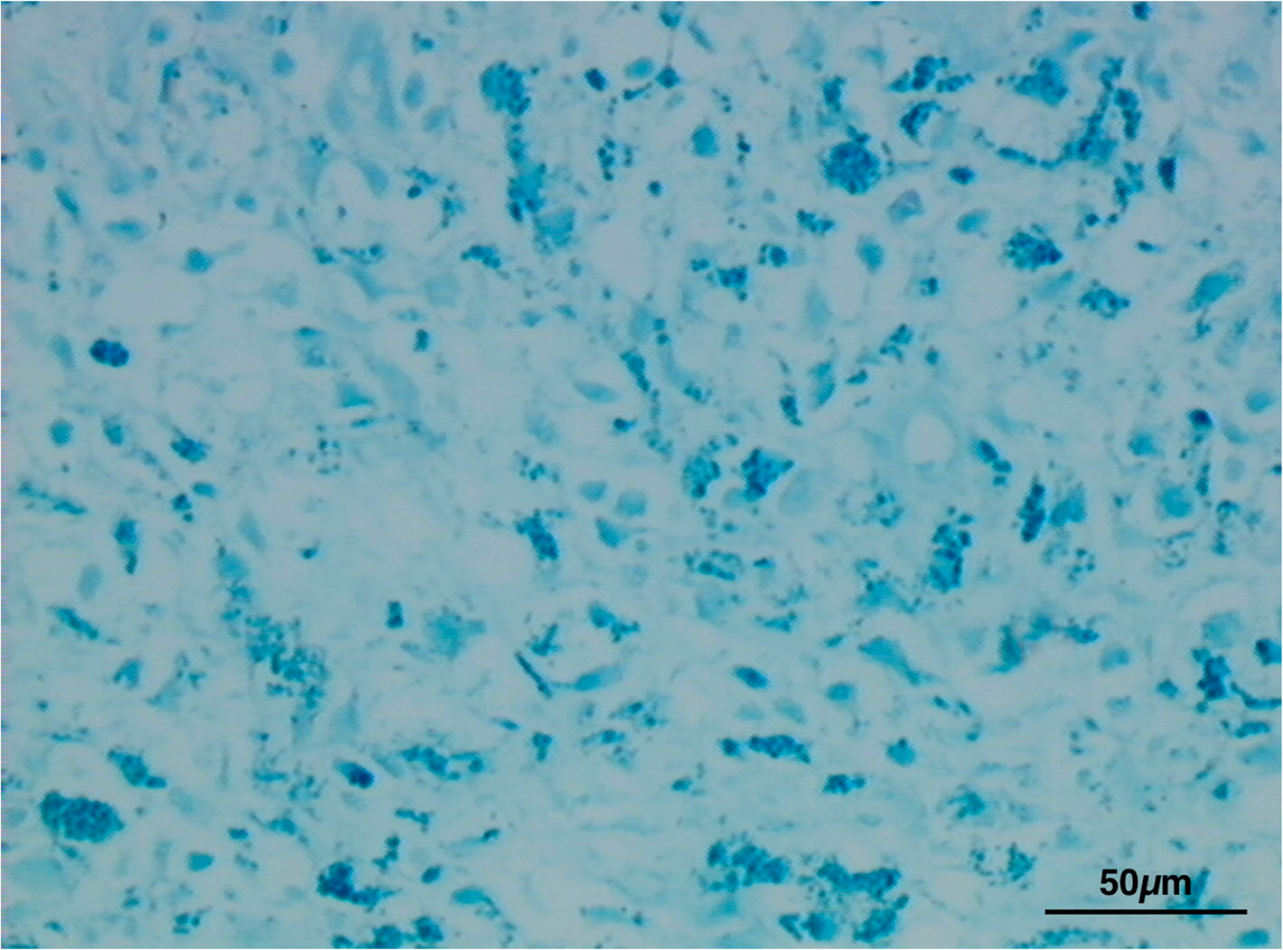
Lymph nodes: Evidence of melanin pigment stained in dark green. (Nile Blue)

**Fig. 4:**
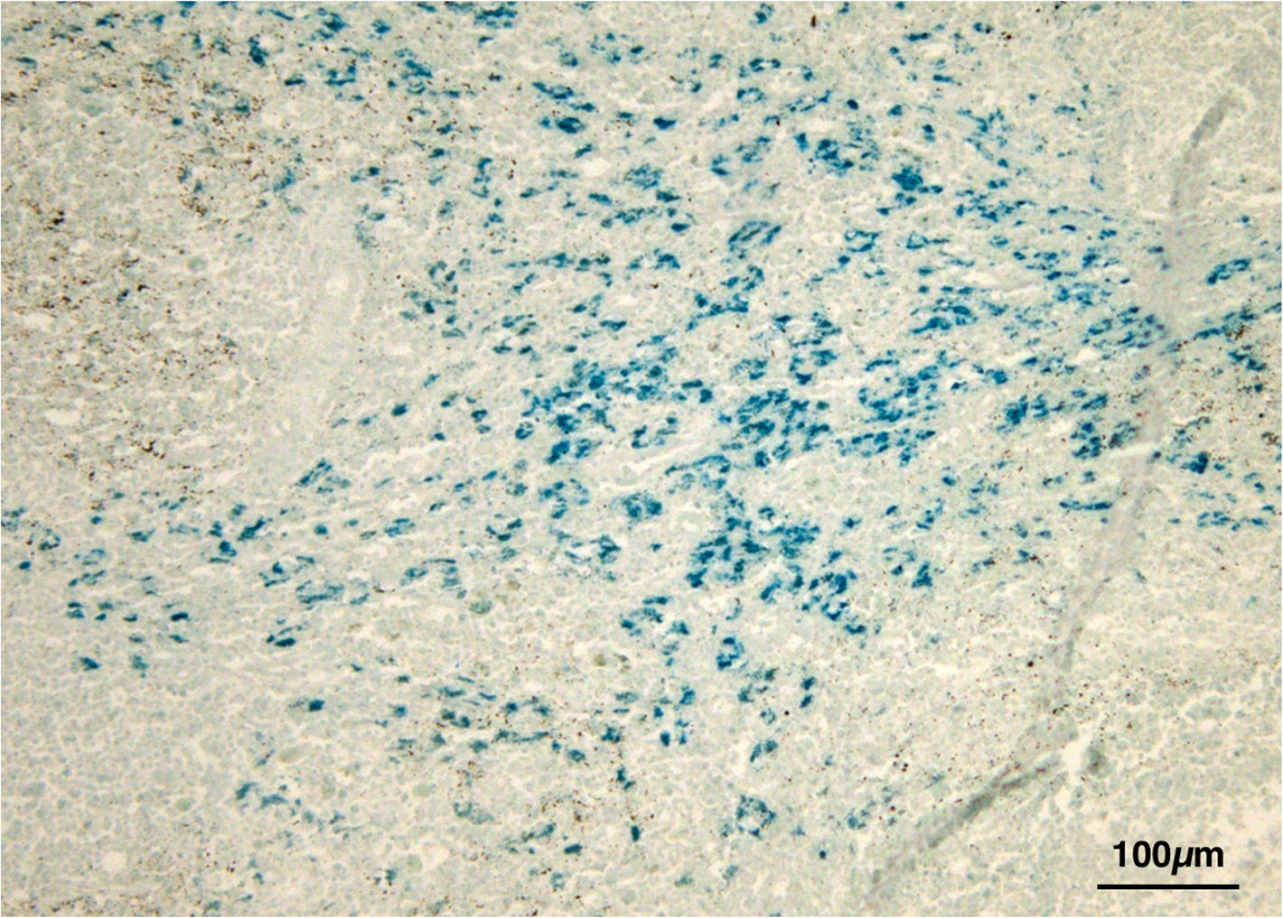
Lymph nodes: Evidence of dark green granules in macrophages. (Lillie)

**Fig. 5:**
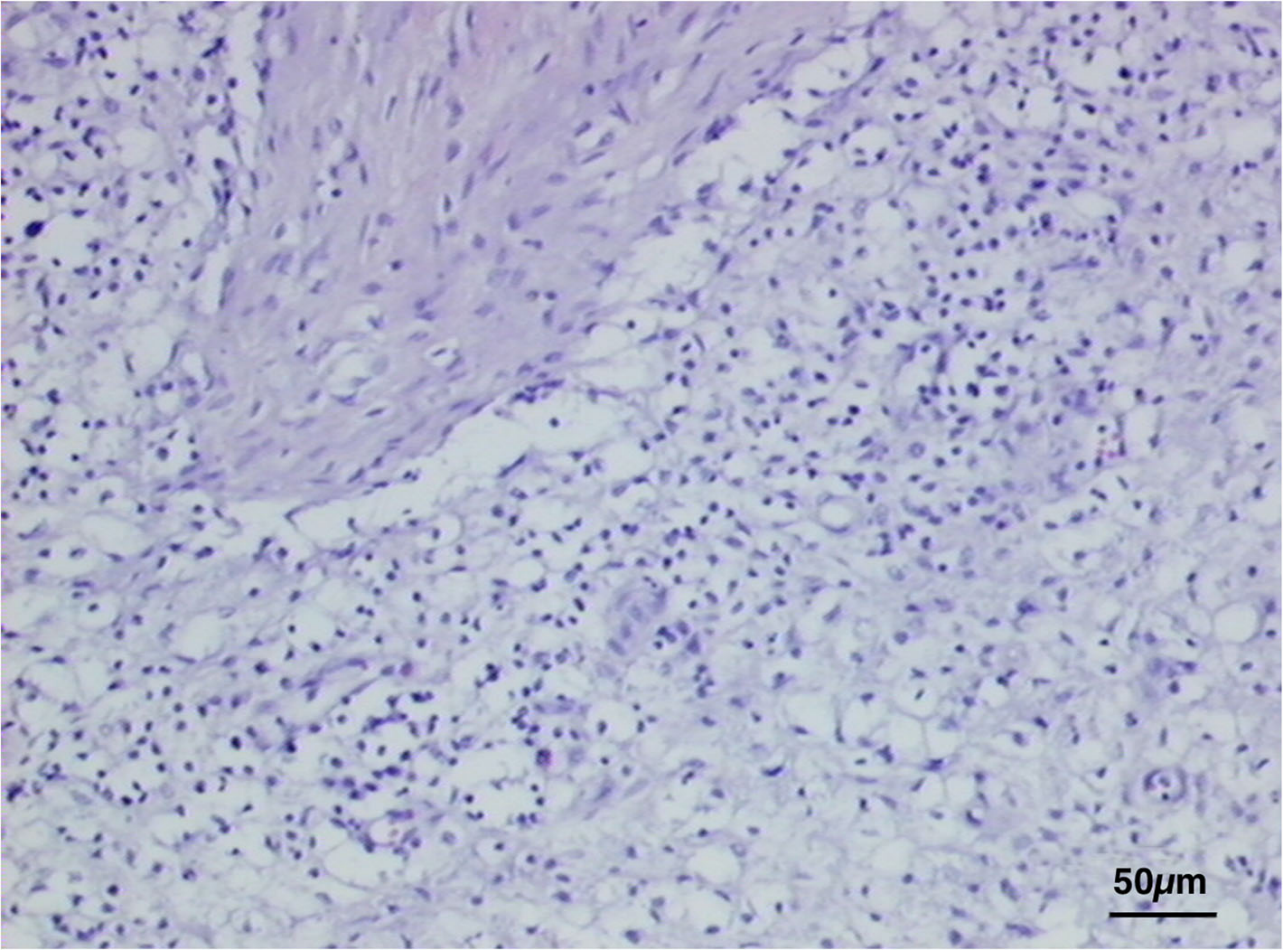
Lymph nodes: Evidence of total disappearance of pigment. (Melanin Bleach)

Histological exam performed on lymph node tissue sections stained with H&E permitted to show a macrophage infiltrate containing a granular brownish pigment from multiple observations (three independent histopathologists) (Fig. 2). Nile Blue elective staining showed a dark green pigment, differentiating it from lipofuscin and thus suggesting a diagnosis of pseudomelanosis (Fig. 3). Finally, Lillie’s method for ferric and ferrous iron (Fig. 4), a highly specific reaction, showed granulation of dark green color in macrophages, providing a further and final confirmation of the diagnosis. Both the two discolorating histochemical tests specific for melanin (Melanin Bleach and Hydrogen Peroxide) revealed the total disappearance of the pigment from the lymph node tissue (Fig. 5).

**Fig. 6:**
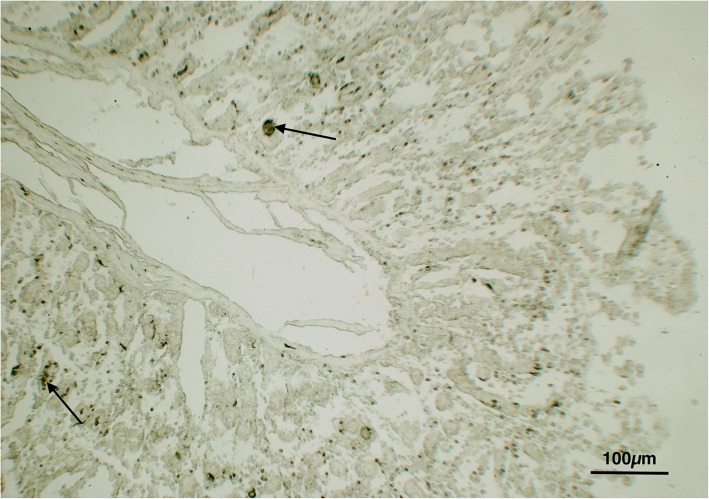
Lymph nodes: Evidence of intracellular pseudomelanin pigment in mastocytes (Arrows) detected in intestinal villi confirming the presence of tyrosinase enzymes at the level of the enteric wall in swine. (Okun’s reaction)

Okun’s reaction, performed on cryostate intestine sections (jejunum), showed formation of intracellular pseudomelanin pigment in mastocytes detected in intestinal villi (Fig. 6), confirming the presence of tyrosinase enzymes at the level of the enteric wall in swine.

No skin melanomas were detected.

**Fig. 7:**
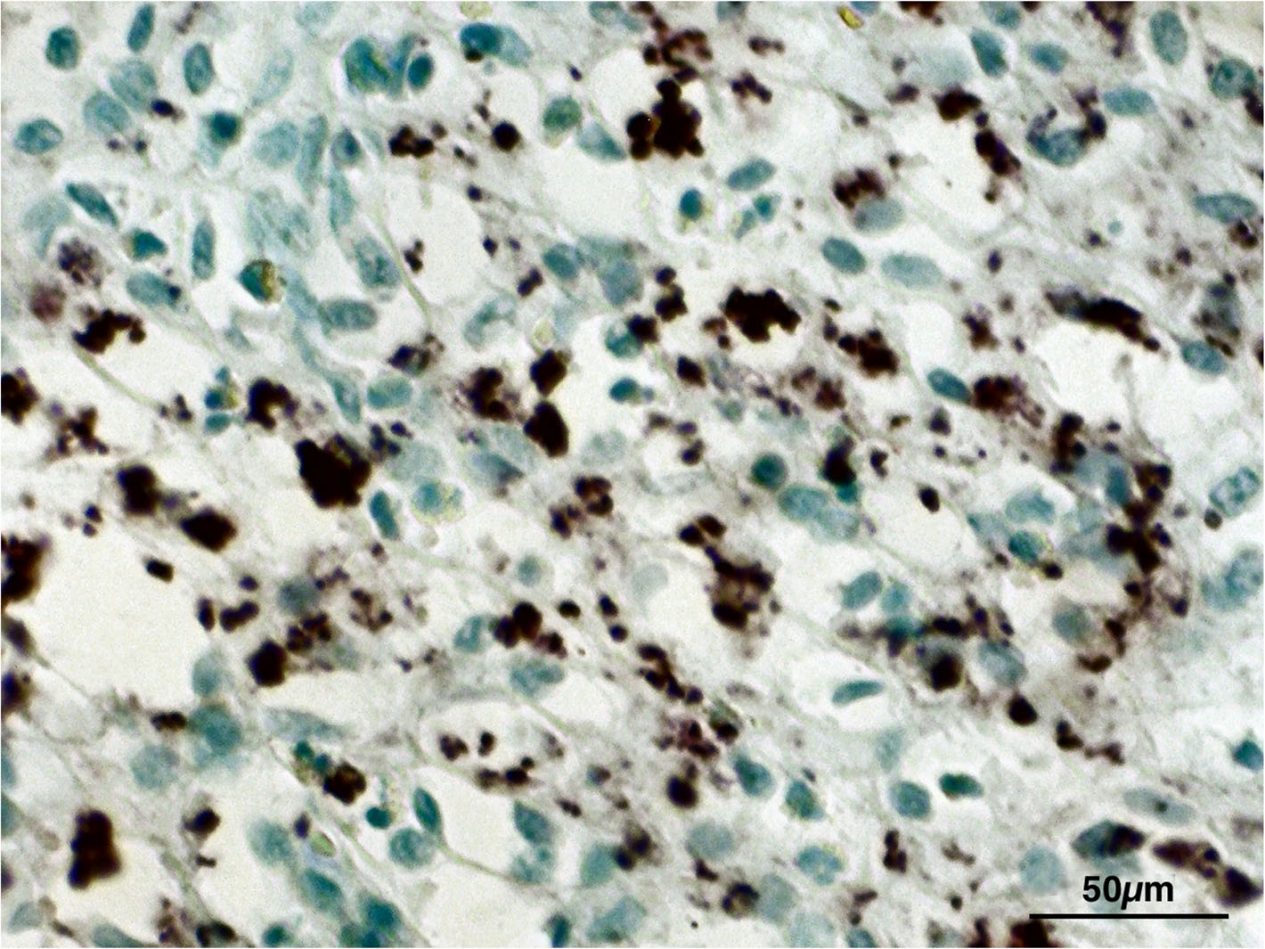
Lymph nodes: Evidence of macrophages containing granular pigment. (Macrophage marker)

The immunohistochemical reaction with macrophage marker (Fig. 7), performed on lymph node tissue sections, showed a strong cytoplasmic expression of macrophages containing variable amounts of pigment. The different concentrations checked for the anti-human melanoma antibody, as well as the immunoreaction against S-100, did not show any expression, thus excluding the hypothesis of melanoma metastasis in lymph nodes.

Results obtained from biochemical assay performed on acorn specimens showed the presence of a higher amount of phenolic substances (mg of gallic acid equivalents/mg of acorn tissue) in Quercus as compared to that of holm oak (Table 2).

**Table 2:** QV: *Quercus virgiliana*; QI: *Quercus ilex*

Acorn	Phenolic Substances
QV	0.0137 ± 0.012 mg
QI	0.0112 ± 0.012 mg

## Discussion

Data here reported represent the first experimental evidence of a feeding related melanosis in *Nero Calabrese pig*. This conclusion is supported by the high incidence of melanosis in acorn fed groups the absence of neoplastic skin lesions, the absence of immunoreaction for anti-human melanoma which cross reactivity has been demonstrated by Lanteri et al. [13], the absence of such pigmentation in control animals, as well as from Okun’s reaction performed on the intestinal mucosa, confirming the acorn related enteric pathogenesis. Thus, the results obtained in this study, considering the well-known process leading to melanin production, permit to hypothesize that the enzyme present at the level of the intestine in swine could start this catalytic process to form melanin, acting on the phenolic substrate of the acorn; moreover, as already shown in Table 1, the higher presence of such substrate in quercus acorn can explain the increased incidence of the pigmentation in those subjects which had been fed this food for a longer period of time/higher quantity. Moreover, groups fed with *Q. virgiliana* showed a greater melanosis prevalence than those fed with *Q. ilex*.

The findings obtained support the feed-related pathogenesis previously suggested also in *Nero Siciliano Pig*, demonstrating for the first time acorn ingestion as cause of acquired melanosis or, rather, a pseudomelanic thesaurismosis in *Nero Calabrese Pig* [4, 12].

Nevertheless, a similar pathogenic possibility was reported as a possible origin of melanosis in pigs fed with acorns [14] even if, considering the limited scientific and technical knowledge at that time, tannins were only suggested as a possible cause although no demonstration was provided.

The role of polyphenol-oxydase and/or peroxydase in pig intestine in activating the phenolic substrate present in acorn, determining the catalysis reaction of the melanin-like pigment, can be stated. Wilberts et al. [15] reported the presence of a melanosis coli in swine due to ceroid pigment storage with multifactorial etiology, studying the association among oxidative damage, protein nitrification and hypovitaminosis E [15].

In human beings, melanosis coli is characterized by a brown-black pigmentation of the colon mucosa and is commonly associated to chronic use of anthracen based laxative (cascara, senna, aloe and rhubarb, among others), containing antrachinone as the active principle. Histologically, melanosis coli is characterized by macrophage carrying pigment in the *lamina propria* of the colon-rectal mucosa [16]. Recent laboratory investigations have shown that the composition of such pigment is not perfectly superimposable with melanin pigment [17]; consequently, the term pseudomelanosis was introduced, suggesting the presence of a melanin-like pigment with exogenous origin. In our opinion, the pathogenesis of the lymph node pigmentations here reported resembles that of melanosis coli, even if in our opinion the term pseudomelanosis should be preferred.

In the field of comparative pathology, the chronic use of vegetable laxatives containing anthraquinone over a long time, in man, was reported to evoke the appearance of melanosis in the colon [17, 18].

## Conclusions

The typical characteristics of rusticity of the *Nero Calabrese Pig*, as well as the *Sicilian* breed, generally farmed in plein-air, make this swine breed an interesting in vivo model to study some disorders which have disappeared or, at least are rare, in modern swine culture, and so it could be considered as a model for comparative study also for some human pathologies.

## Methods

In this study, 142 pigs belonging to the “Nero Calabrese” breed, both males and females, of about 18–24 months in age, reared in plein-air in a forest area of Aspromonte and fed with natural foods derived from the environment, with a supplement of commercial food in different percentages depending on seasonal availability in the territory were analyzed. All the animals belong to the “Strangio” farm and were selected on the basis of a total absence of melanoma lesions, such as maculae, nodules or exophytic masses; moreover, because of continuous meat production animals were randomly selected throughout the year, permitting to evaluate the effect of different acorns on lymph node discoloration. Pigs were subdivided into 5 groups and were regularly slaughtered at the public slaughterhouse of Siderno (RC), in the period 2015–16:

1. Group A: n° 34 subjects, 70% natural fed/present in grazing [quercus acorn “*Quercus virgiliana*” (crude protein 4.2%; etheric extract 2.9%; crude fiber 2.6%), roots, tubers] + 30% commercial fed (barley, field bean, maize, wheat feed, citrus fruit essential pulps, carob beans, sugar cane molasses, calcium carbonate, bi-calcic phosphate, sodium chloride. Analytic Composition: crude protein 17%; etheric extract 3.4%; crude fiber 4.6%, ash 5.7%).
2. Group B: 42 subjects, 40% natural fed/present in grazing [quercus acorn “*Quercus virgiliana*” (crude protein 4.2%; etheric extract 2.9%; crude fiber 2.6%), roots, tubers] + 60% commercial fed (barley, field bean, maize, wheat feed, citrus fruit essential pulps, carob beans, sugar cane molasses, calcium carbonate, bi-calcic phosphate, sodium chloride. Analytic composition: crude protein 17%; etheric extract 3.4%; crude fiber 4.6%, ash 5.7%).
3. Group C: 35 subjects, 70% natural fed/present in grazing [holm oak acorn “*Quercus ilex*” (crude protein 4.6%; etheric extract 2.8%; crude fiber 2.8%), roots, tubers] + 30% commercial fed (barley, field bean, maize, wheat feed, citrus fruit essential pulps, carob beans, sugar cane molasses, calcium carbonate, bi-calcic phosphate, sodium chloride. Analytic composition: crude protein 17%; etheric extract 3.4%; crude fiber 4.6%, ash 5.7%).
4. Group D: 31 subjects, 40% natural fed / present in grazing [holm oak acorn “*Quercus ilex*” (crude protein 4.6%; etheric extract 2.8%; crude fiber 2.8%), roots, tubers] + 60% commercial fed (Composition: barley, field bean, maize, wheat feed, citrus fruit essential pulps, carob beans, sugar cane molasses, calcium carbonate, bi-calcic phosphate, sodium chloride. Analytic composition: crude protein 17%; etheric extract 3.4%; crude fiber 4.6%, ash 5.7%).
5. Group E: 29 subjects, 100% commercial fed (barley, field bean, maize, wheat feed, citrus fruit essential pulps, carob beans, sugar cane molasses, calcium carbonate, bi-calcic phosphate, sodium chloride. Analytic composition: crude protein 17%; etheric extract 3.4%; crude fiber 4.6%, ash 5.7%).

Sample size was determined on the basis of the number of pigs actually reared on the farm in a one-year period. Due to the homogeneity of the sample size in the different groups, all animals were considered for statistical evaluation.

At regular slaughtering, all organs were examined. Lymph nodes and intestine (jejunum), were sampled and photographed; parts of the tissue samples were fixed in 10% formalin solution and embedded in paraffin wax. Tissues coming from 10 specimens for each experimental group were used for histological, histochemical and immunohistochemical analysis. 5 μm thick paraffin embedded sections were routinely stained with haematoxilin-eosin (H&E).

Histochemical evaluation was performed on deparaffinized histological sections to identify the cell types involved and to characterize the pigment stored, using the following staining and bleaching methods:

### Histochemical staining reactions

Nile Blue (hydrogen sulfate): elective staining method based on hydrogen sulfate specific to differentiate neutral fat and cholesterol esters from free fat acids and from phospholipids; such reaction permits to differentiate melanin pigment from lipofuscin [19]. Lillie’s method: this histochemical reaction, based on ferric sulfate and potassium iron cyanide, is highly selective for melanin, showing pigment granules stained in dark green [20]. Okun’s method: elective histochemistry for tyrosinase detection in intestine, through catalysis of the L-tyrosine and DL-DOPA in melanin. This reaction, performed on cryostated sections, is severely damaging for tissue sections [21]. Bleaching histochemical methods. Hydrogen peroxide: histological sections are treated for 24–48 h with 10% H_2_O_2_. After this treatment, melanin present in tissues, both externally and in macrophages, is discolored. Melanin is the only pigment that bleaches with this reaction [2]. Melanin bleach: this method is based upon the same principle of bleaching using hydrogen peroxide but using potassium permanganate and oxalic acid [2].

### Immunohistochemistry staining reactions

To further confirm the pigmentation disorder, eliminating every doubt on the possible pathogenetic hypothesis of melanosis subsequent to regressive congenital melanoma, the following immunohistochemical stains were carried out on 10 μm thick histological sections, developed from an Avidin-biotin complex (BioSpa, 20,143, Milano, Italy) and revealed by DAB (Diaminobenzidine, Vector Laboratories, Inc. U.S. Headquarters, Burlingame, California, USA): Macrophage marker (Novocastra, Newcastle Upon Tyne, UK, Product Code NCL-MAC387; dilution 1:100), mouse monoclonal antibody with high specificity towards leucocyte antigens and L1 proteins, important for macrophage, monocyte and histiocyte labelling; to differentiate melanin pigmentation from immunohistochemistry labelling, sections were bleaching for 150 min by using 10% H2O2 in phosphate-buffered saline (PBS) at 60C° as proposed by Orchard [22]. Monoclonal mouse anti-human melanoma (Dako, Denmark, Product Code M 7258; dilution 1:25, 1:50, 1:100, 1:200), specific against the Melan A antigen; this antibody, provided for use in immunocytochemistry and immunohistochemistry, permits to show antigens at 45, 68, 75 KDa (positivity range known for pig) on the basis of concentration [23]. Exams were performed in duplicate with two different rates, on lymph node tissue sections. Polyclonal rabbit anti-S100 (Dako, Denmark, Product Code Z 0311; dilution 1:1000), antibody labelling normal melanocytes, Langerhans cells, histiocytes, condrocytes, adipocytes, cardiac and skeletal muscle, Schwann cells, epithelial and myoepithelial mammary cells, salivary and sweat glands, and glial cells. For each sample, negative controls were also performed by omission of primary antibody (Ab) and substitution of primary antibody with an indifferent rabbit primary antibody (Additional files 2, 4). Moreover, melanoma tissue obtained from a Nero Siciliano Pig was used as positive control [13] (Additional files 1, 3).

### Biochemical reactions

Total phenolic substances were identified from acorns through the use of a methanolic extract with Folin-Ciocalteau reagents [24]; data obtained revealed the total amount of phenolic substances expressed as amount of gallic acid on mg of acorn tissue.

### Statistical analysis

Chi square analysis was performed to evaluate difference in pseudomelanosis prevalence among the Groups. *P* values < 0.05 were considered statistically significant.

Data were analyzed using statistical software Prism v. 4.00 (Graphpad Software Ldt., USA, 2003).

## Additional files

Additional file 1: Melan A: positive control in melanoma from pig (bar 50 μm). (TIF 4973 kb)

Additional file 2: Melan A: negative control from pig lymph node, obtained by omission of the primary antibody (AB) and substitution of primary antibody with an indifferent rabbit primary antibody (bar 50 μm). (TIF 3738 kb)

Additional file 3: S100: positive control in melanoma from pig (bar 50 μm). (TIF 3470 kb)

Additional file 4: S100: negative control from pig lymph node, obtained by omission of the primary antibody (AB) and substitution of primary antibody with an indifferent rabbit primary antibody (bar 100 μm). (TIF 3500 kb)

## Data Availability

The datasets used and analyzed during the current study are available from the corresponding author on reasonable request.

## Abbreviations

DAB: Diaminobenzidine; KDa: Kilodalton
DL-DOPA: D-form of Dihydroxyphenylalanine
FAO: Food and Agriculture Organization
H&E: Haematoxylin and eosin
RC: Reggio Calabria
