# Supplementary figures and images for: Acorn-related acquired pseudomelanosis in Calabrian black pigs
Source: BMC Vet Res. 2019 Jun 4;15:186. doi: 10.1186/s12917-019-1934-5 (PMC6549356; doi:10.1186/s12917-019-1934-5)

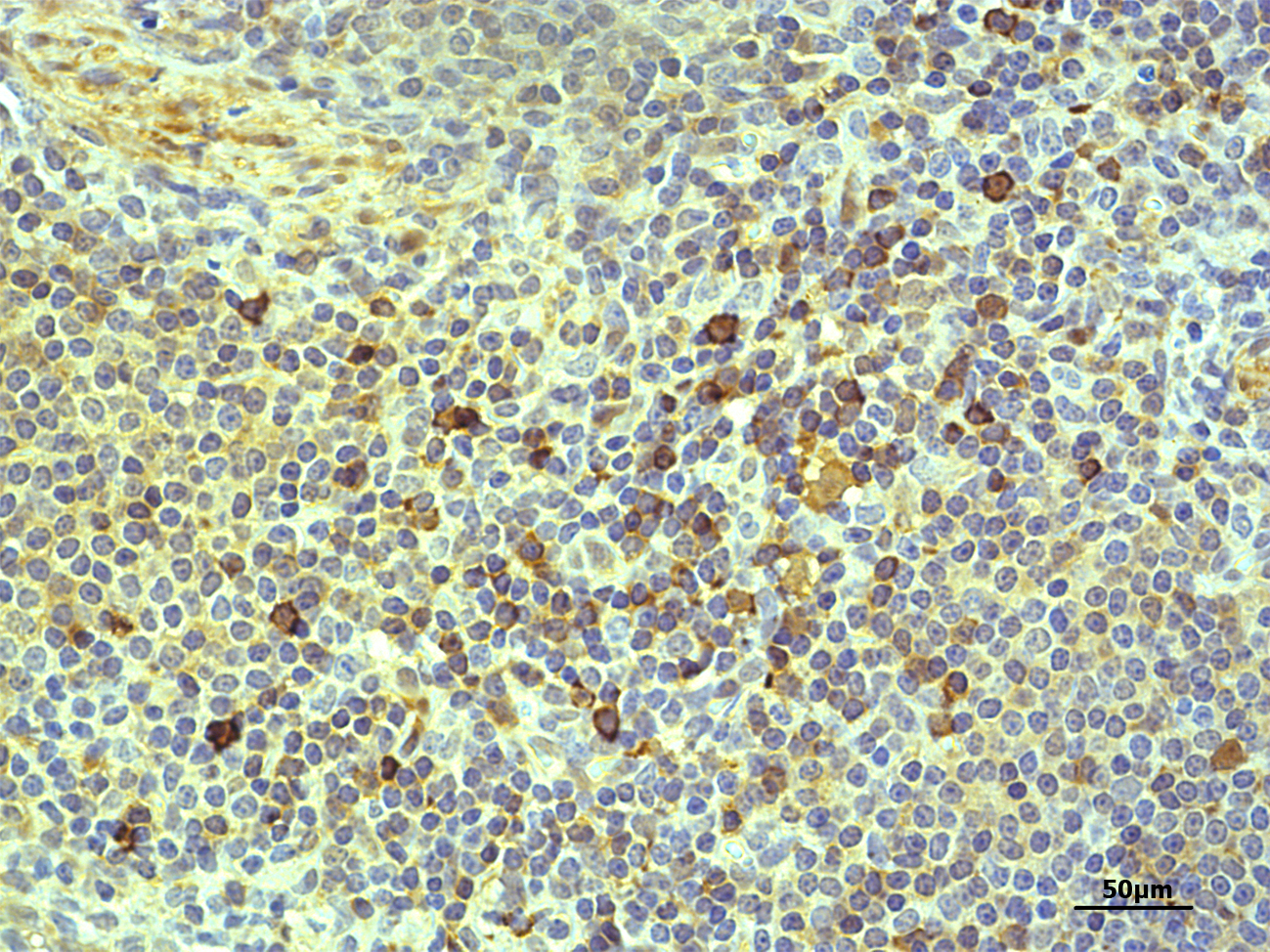

Supplement: Supplementary file 1 — Melan A: positive control in melanoma from pig (bar 50 μm). (TIF 4973 kb) [file 12917_2019_1934_MOESM1_ESM.tif]

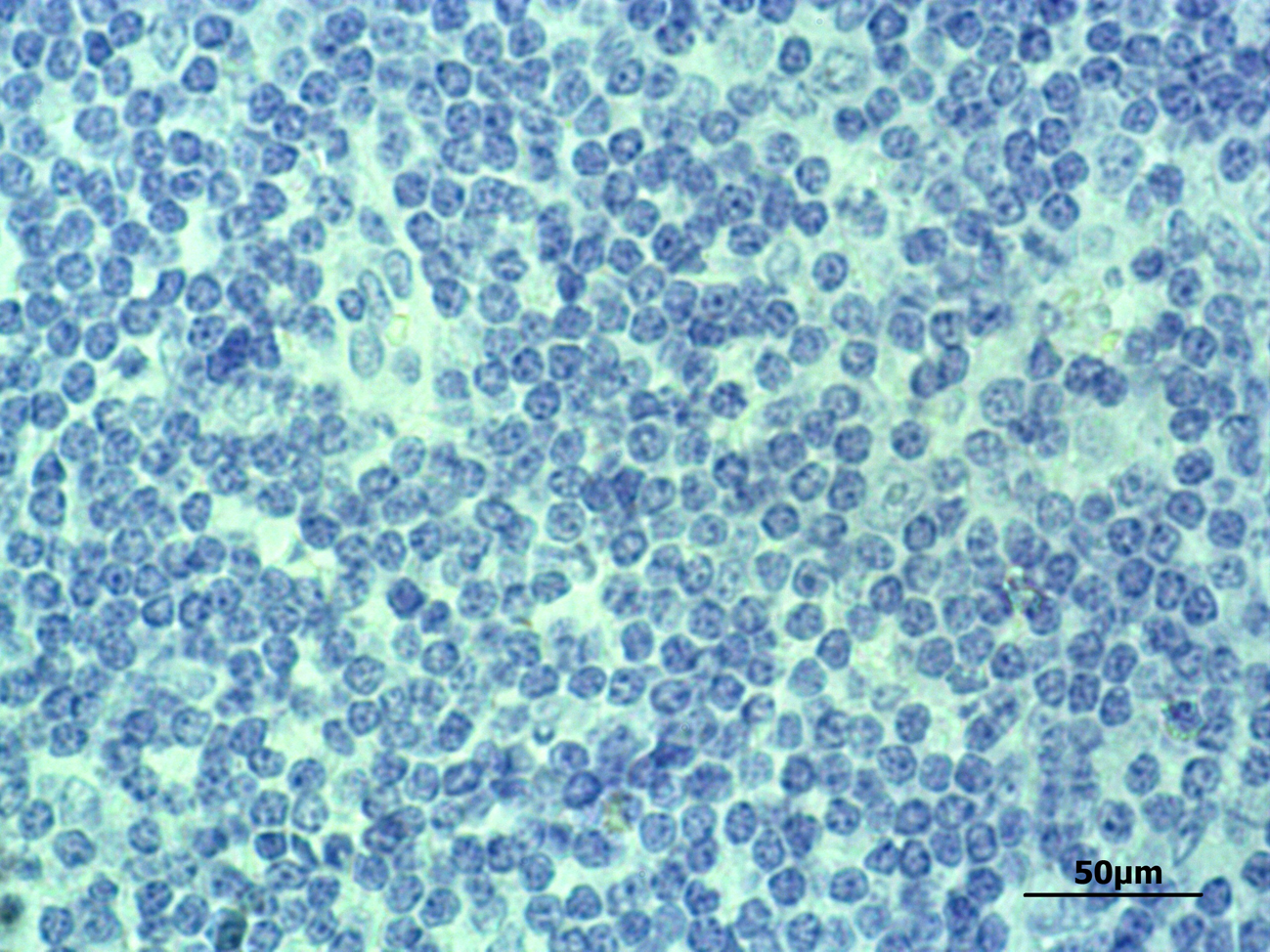

Supplement: Supplementary file 2 — Melan A: negative control from pig lymph node, obtained by omission of the primary antibody (AB) and substitution of primary antibody with an indifferent rabbit primary antibody (bar 50 μm). (TIF 3738 kb) [file 12917_2019_1934_MOESM2_ESM.tif]

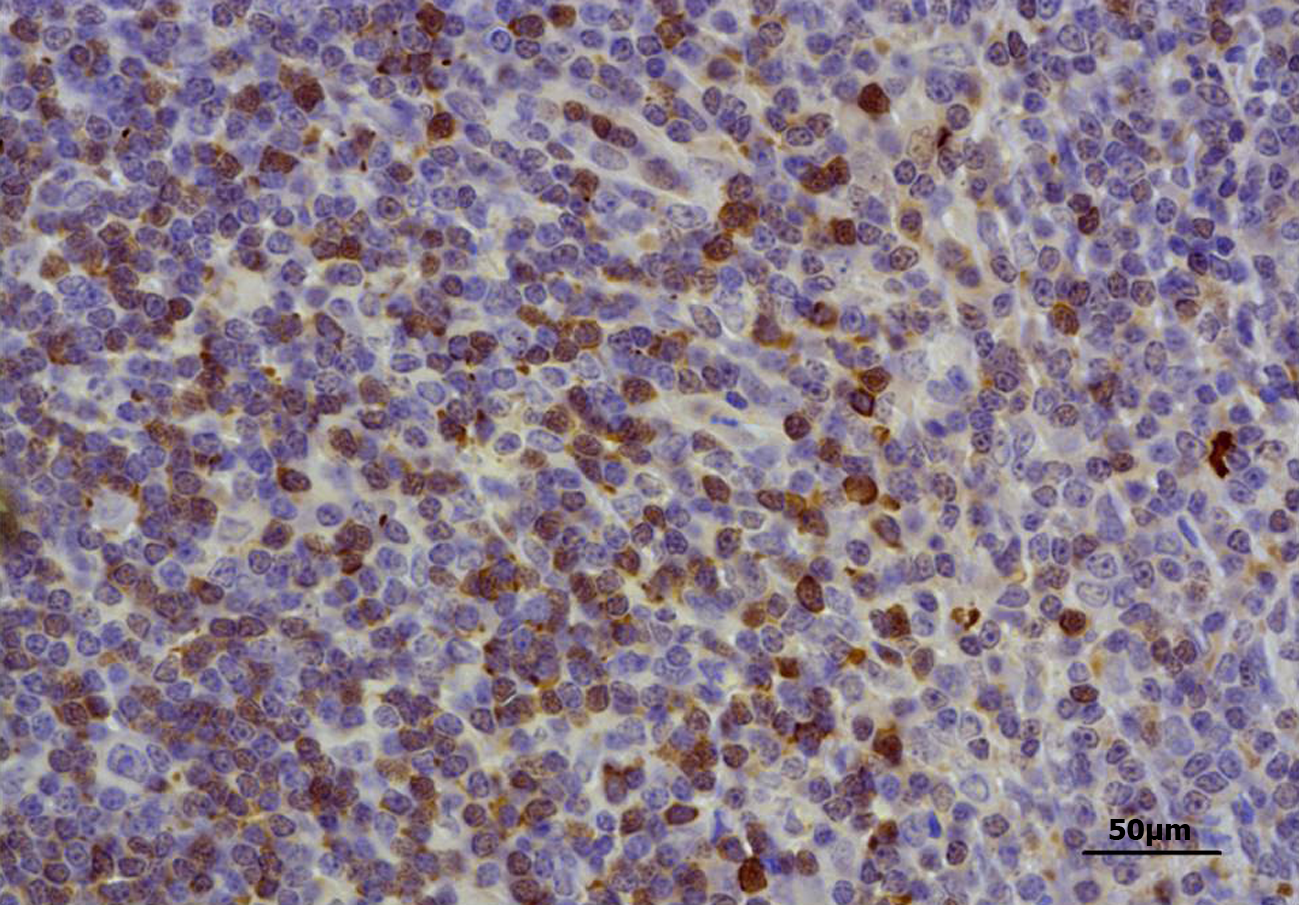

Supplement: Supplementary file 3 — S100: positive control in melanoma from pig (bar 50 μm). (TIF 3470 kb) [file 12917_2019_1934_MOESM3_ESM.tif]

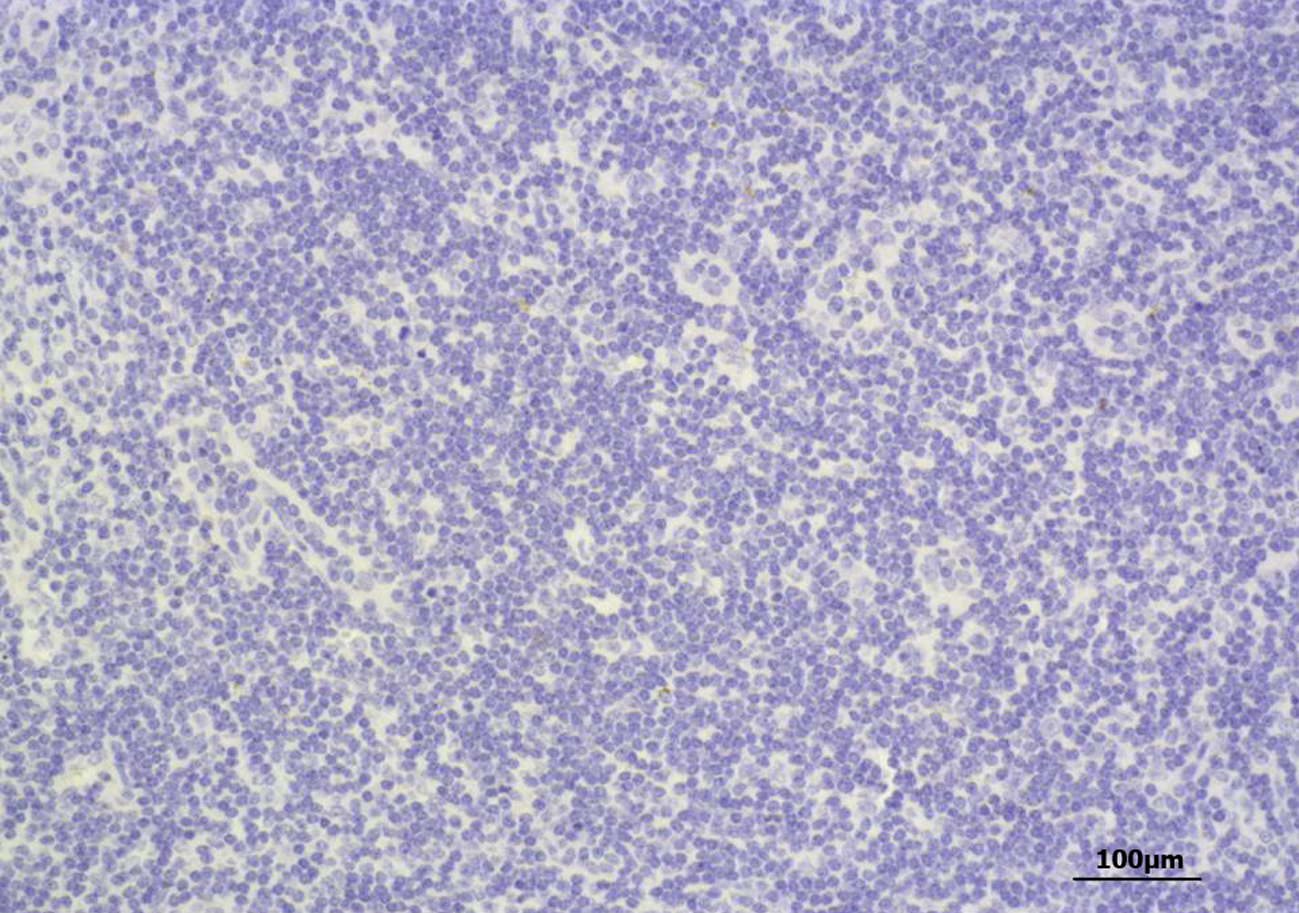

Supplement: Supplementary file 4 — S100: negative control from pig lymph node, obtained by omission of the primary antibody (AB) and substitution of primary antibody with an indifferent rabbit primary antibody (bar 100 μm). (TIF 3500 kb) [file 12917_2019_1934_MOESM4_ESM.tif]
